# Low Levels of HDL in Fragile X Syndrome Patients

**DOI:** 10.1007/s11745-015-4109-6

**Published:** 2015-12-28

**Authors:** Małgorzata Z. Lisik, Ewa Gutmajster, Aleksander L. Sieroń

**Affiliations:** Department of Molecular Biology and Genetics, School of Medicine in Katowice ul., Medical University of Silesia, ul. Medyków 18, 40-752 Katowice, Poland

**Keywords:** Lipid biochemistry, General area, HDL, Lipoproteins, LDL, Lipoproteins

## Abstract

Fragile X syndrome (FXS) is the most common form of familial mental retardation and one of the leading known causes of autism. The mutation responsible for FXS is a large expansion of the CGG repeats in the promoter region of the *FMR1* gene resulting in the transcriptional silencing of the gene in the pathophysiology of Fragile X syndrome was hypothesized. 23 male patients affected by Fragile X syndrome (full mutation in the *FMR1* gene) and 24 controls were included in the study. The serum levels of HDL-C were lower in FXS patients (*p* < 0.001). The serum levels triacylglycerols were higher in FXS patients (*p* = 0.007) Further study involving larger samples are necessary to confirm the results and define the health implications for abnormal lipid levels in FXS patients.

## Introduction

Fragile X syndrome (FXS, MIM # 309550), is the most common inherited form of mental retardation. It has an estimated frequency of 1/7000 males and 1/11,000 women [1]. Affected males display varying degrees of symptoms ranging from mild to severe [2]. The behaviour of boys with FXS typically includes attention deficit hyperactivity disorder (ADHD) with significant impulsivity and anxiety, as well as repetitive language and hand stereotypes. Specific behaviour combined with social and language deficits, often lead to a diagnosis of autism spectrum disorder (ASD) before the FXS is diagnosed [2]. The Fragile X syndrome is the most common single genetic cause of autism, occurring in 1–6 % of boys with ASD [3]. In almost all cases the disease is caused by an expansion of the unstable CGG repeat sequence located in the 5′ untranslated region of the *FMR1* gene [4]. In affected individuals the CGG repeat length exceeds 200 and is called the full mutation (FM). The promoter pf the *FMR1* gene is usually hypermethylated and the methylation involves also the adjacent promoter region of the *FMR1* gene. The gene is transcriptionally silenced and the gene product, the Fragile X mental retardation protein (FMRP), is absent [5].

Loss of FMRP, an RNA binding protein, which is a negative regulator of group I mGluR results in increased synthesis of proteins normally regulated by FMRP, dysregulation of synaptic signal transduction and channel function, excess mGluR signalling and dysfunction of synaptic plasticity [6].

A spectrum of medical problems in fragile X syndrome patients has been reported, such as seizures, recurrent otitis media, gastrointestinal disturbances. The behavioural phenotype of FXS includes significant anxiety, poor eye contact, attention deficit hyperactivity, hyperarousal to sensory stimuli, irritability, repetitive motor behaviors. Ocular disorders are common in FXS patients. Many of them need glasses because of refractive errors. Sleep problems are observed in 10–25 % of children and adolescents. Loud snoring and obstructive sleep apnoea occurred in 38 and 34 % of children with FXS [7].

We found only one study describing cholesterol levels in adult individuals with FXS. The Berry-Kravis study in [8] described cholesterol levels in both children and adults.

To confirm and extend these findings, we prospectively measured serum levels of total cholesterol (TC), high-density cholesterol fraction (HDL-C), low-density cholesterol fraction (LDL-C) and triacylglycerols, in 23 males with FXS and 24 age-matched controls.

## Study Participants and Methods

The study was conducted at the Department of Molecular Biology and Genetics, Silesian Medical University School of Medicine in Katowice, from February to May 2012.

Twenty three male patients, mean age 19.3 ± 6.6, affected by Fragile X syndrome, confirmed by detection of full mutation in the *FMR1* gene, were recruited through registry databases maintained by Medical University of Silesia, Upper Silesia Center of the Children’s Health John Paul II in Katowice. All participants harboured the “full mutation” form of the *FMR1* gene. Diagnosis of Fragile X syndrome was based on clinical criteria and molecular analysis of the *FMR1* gene. The control group comprised 24 healthy males, mean age 21.8 ± 5.8 years. The study protocols were approved by the local Ethical Committee of the Faculty of Medicine, Medical University of Silesia in Katowice. In addition, an informed written parental/legal guardian consent was obtained from the parents/legal guardians of the studied subjects.

A complete medical evaluation, including medical history, family history, psychological testing and physical examination were conducted for each subject. The patients and controls were not taking any lipid-modifying drugs.

Venous blood samples were drawn from each subject by venipuncture into a sodium heparin tube between 8 and 10 am after having been instructed to fast the previous 12 h. Then the blood was centrifuged at 3000×*g* for 10 min. Plasma was collected and stored at −80 °C until further study.

Serum total cholesterol (TC), triacylglycerols, high-density cholesterol fraction (HDL-C), low-density cholesterol fraction (LDL-C) were determined in all the study participants. Triacylglycerols, TC and HDL-C were measured by an enzymatic colorimetric method using the BM Roche/Hitachi 717 analyzer (kits of Roche). LDL-C was calculated using the formula LDL-C = TC − HDL-C − triacylglycerols/5 [9].

### Statistical Analysis

Data are presented as means ± standard deviations. The Kolmogorov–Smirnov test was used to determine whether continuous variables are normally distributed. Differences between normally distributed variables were assessed with the independent samples *t* test. The Mann–Whitney *U* test was used to examine all non-normal continuous variables and Spearman’s non-parametric correlation coefficient to calculate correlations. For all tests, a probability (*p*) of less than 0.05 was considered statistically significant. Statistical analyses were performed with SPSS statistics software, version 10 (StatSoft. Inc. DELL, USA).

## Results

Patients and controls were matched for age. The demographic and anthropometric data are shown in Table 1. Serum lipid profiles are shown in Table 2. The results are presented as means and standard deviations. Overweight and obesity were observed in 60.86 % of FXS group.

**Table 1:** Dermographic characteristics of patients with FXS and controls

	FXS group		Control group		*p*
	X ± SD	Range	X ± SD	Range	
Age (years)	19.3 ± 6.6	10–32	21.8 ± 5.8	8–29	0.07
Body mass (kg)	77.6 ± 21.1	28.0–103.5	71.5 ± 19.4	22.0–94.0	0.1
Height (cm)	170 ± 15	120–150	173 ± 17	119–192	0.1
BMI	26.5 ± 4.7	18.5–34.9	22.9 ± 3.4	14.9–27.7	0.01

**Table 2:** Serum lipid profiles of patients with FXS and controls

	FXS group		Control group		*p*
	X ± SD	Range	X ± SD	Range	
Cholesterol (mg/dl)	157.9 ± 39.1	113.7–311.9	170.4 ± 36.4	107.9–229.9	0.09
HDL (mg/dl)	43.4 ± 7.0	32.3–58.6	56.3 ± 12.6	36.9–103.2	<0.0001
LDL (mg/dl)	97.8 ± 35.5	41.9–225.3	105.1 ± 30.8	46.3–164.5	0.2
Triacylglycerols (mg/dl)	133.3 ± 57.2	51.0–257.5	94.7 ± 64.6	24.7–264.0	0.035
HDL/LDL	0.488 ± 0.187	1.103–0.257	0.596 ± 0.31	1.734–0.373	0.1596

Comparison of the TC, HDL, LDL and triacylglycerols, values from FXS patients to those from the control group showed that mean values of the serum levels of HDL-C were significantly lower in FXS patients (43.4 ± 7.0 FXS *vs* 56.3 ± 12.6 control, *p* < 0.001), the serum levels of triacylglycerols, were higher in FXS patients (133.3 ± 57.2 FXS *vs* 94.7 ± 64.6 controls, *p* = 0.035). The TC and LDL-C levels did not differ statistically from the controls. There was no significant correlation between BMI and TC, HDL-C or LDL-C levels in FXS group. HDL/LDL did not differ statistically.

## Discussion

This study analysed the serum levels of total cholesterol (TC), triacylglycerols, high-density cholesterol fraction (HDL-C), low-density cholesterol fraction (LDL-C) in FXS patients and used an age-matched control group. Results indicated that the serum levels of HDL-C were lower in FXS patients and serum levels of triacylglycerols were higher in FXS patients compared to the control group. HDL-C was more significantly reduced relative to the controls. The TC and LDL-C levels were lower in the FXS group, but the difference was not statistically significant.

Berry-Kravis *et al*. [8] performed a chart review to collect information on cholesterol levels from patients with FXS attending the clinic program. This retrospective study strongly suggested that TC, HDL-C and LDL-C levels were lower in males with FXS than normative population data. Our prospective study confirmed the previously reported low HDL-C in FXS but was not powered to detect a significant difference in TC and LDL-C because the amount of difference was less than for HDL-C.

There was no significant correlation between BMI and TC, HDL-C or LDL-C levels in FXS group. Our observations confirmed the lack of relationship between BMI and lipid levels in FXS observed by Berry-Kravis *et al*. [8].

HDL-C are remarkably complex, dynamic and heterogeneous particles defined by the density range 1063–1.21 g/ml. Serum levels of HDL-C are inversely related to risk of atherosclerotic cardiovascular disease. Among patients with premature cardiovascular disease, low HDL-C is the most common lipid abnormality. However, data from recent clinical trials, which used various methods to raise HDL-C levels have not showed the expected improvement in CVD. HDL-C has long been known to have anti-inflammatory properties, but during the acute inflammatory response, HDL changes to a pro-inflammatory state [10].

The observation that the serum levels of HDL-C are lower and serum levels of triacylglycerols, are higher in FXS patients has implications for health monitoring and treatment practices. The low HDL-C may be risk factor for development of vascular and/or cardiac disorders. According to the guidelines for health supervision of patients with FXS growth and weight parameters, auscultation of the heart and blood pressure values should be obtained at clinic visits. Standard monitoring of lipid profiles in FXS patients should be also involved in health supervision [11].

FMRP is an RNA-binding protein that regulates stimulus-dependent translation of a large number of proteins. Misregulation of one or many proteins which in normal situation are regulated by FMRP may be responsible for the lower HDL and higher TG observed in FXS patients. The recent analysis of the genes involved in lipid homeostasis revealed that several FMRP target genes are linked to regulation of lipid levels, including APOE, GSK3B [12–14]

This study is limited by a relatively small sample size. The nearly significant difference between the age of the FXS and control group may be a limitation of the study. The lipid levels vary with age, especially in children and adolescents. Further studies are needed to confirm the results in a larger prospective study group. Further study involving larger samples are necessary to confirm the results and define the health implications for abnormal lipid levels in FXS patients.

## Abbreviations

ADHD: Attention deficit hyperactivity disorder
ASD: Autism spectrum disorder
FM: Full mutation
FMRP: Fragile X mental retardation protein
FXS: Fragile X syndrome
HDL-C: High density cholesterol fraction
LDL-C: Low density cholesterol fraction
TC: Total cholesterol
